# Physical Activity and Recurrent Pain in Children and Adolescents in Germany—Results from the MoMo Study

**DOI:** 10.3390/children9111645

**Published:** 2022-10-28

**Authors:** Simon Kolb, Alexander Burchartz, Laura Krause, Leon Klos, Steffen C. E. Schmidt, Alexander Woll, Claudia Niessner

**Affiliations:** 1 Institute of Sports and Sports Science, Karlsruhe Institute of Technology, 76131 Karlsruhe, Germany; 2 Robert Koch Institute, Department of Epidemiology and Health Monitoring, 12101 Berlin, Germany

**Keywords:** headache, abdominal pain, back pain, motor performance, physical fitness, KiGGS

## Abstract

Recurrent pain can be a significant disruption in the activities of daily life, and is not only a health problem in adults but also in children and adolescents. This study analyzed the prevalence of recurrent pain in the current sample (n = 1516; 11–17 years (mean_age_ = 14.4 ± 2.0 years); 50.8% female) of a nationwide study in Germany, evaluated the association of participants’ device-based physical activity (PA) with the prevalence of recurrent pain, and assessed whether children and adolescents who reported pain for the last three months accumulated less PA than those who did not. A higher prevalence was found in girls for recurrent headaches (42.2% vs. 28.7%), abdominal pain (28.2% vs. 20.1%), and back pain (26.9% vs. 19.5%). We found higher odds for recurrent headaches in girls (OR = 1.54) and in participants that did not reach at least 60 min of moderate to vigorous PA (MVPA) per day (OR = 2.06). Girls who reported recurrent headaches accumulated 4.7 min less MVPA per day than those without. The prevalence of pain remains at a high level in the German youth and underscores the need for interventions to improve the health situations of children and adolescents.

## 1. Introduction

Recurrent pain is not only a health problem in adults, but also in children and adolescents [1,2,3,4], and can cause significant disruptions in daily activities [3,4,5]. Affected children have more absences from school and participate less frequently in athletic or social activities [3,5]. Furthermore, the utilization of medical care and medications increases in this group [6,7,8]. Additionally, recurrent pain has been shown to affect well-being. This can manifest itself, for example, in a reduced quality of life (HRQoL) or emotional problems such as anxiety and depressiveness [7,9].

Although the scientific evidence is limited, PA and especially aerobic activities are regularly recommended to migraine patients [10,11]. The results of a study by Pilati et al. showed a protective effect in women and support the protective role of exercise with regard to migraines [12]. Others found moderate evidence that aerobic exercise reduces the number of migraine days, but only low evidence for decreased durations of the attacks and intensity of pain [13]. In a qualitative interview study, low-intensity PA was mentioned by adolescents to be helpful in coping with headaches [14]. However, in another study, PA did not show a predictive function for any of the studied headache disorders [15]. A meta-analytic review of the hypoalgesic effects of exercise revealed highly variable magnitudes and directions of the effects in the chronic pain group, depending on the pain condition and the intensity of the exercise [16]. A possible mechanism to explain exercise-induced hypoalgesia is the release of peripheral and central beta-endorphins during exercise, which influence pain sensitivity [16]. In addition to the neurovascular processes, other cardiopulmonary as well as inflammatory processes come into question to explain how improved aerobic fitness affects pain and the perception of it [17].

Part of the conflicting results may be due to the fact that a single dose of physical activity can trigger pain or can cause a pain condition to worsen, while regular physical activity can help relieve pain by increasing serotonin levels and increasing opioids in the central inhibitory pathway [18]. 

Another point that must be considered in connection with the different results is the recording of PA. Most of the studies assessed PA via self-reports. Some studies ask about the number of days of physical activity without considering duration, type, and intensity [19]. Other studies, especially in the field of therapy, are based on a specific exercise program, the effectiveness of which is tested. In principle, different types and intensities of physical and sporting activity have different effects on the body, and therefore different effects on pain conditions or the perception of pain are to be expected. 

Moreover, PA has also been found to be beneficial in the treatment of several conditions commonly associated with migraines (e.g., obesity, hypertension, and depression) [17]. Obesity and low levels of PA also demonstrated an independent as well as a combined association with recurrent headaches in adolescents [19].

Numerous studies from different European countries and other parts of the world have shown an increasing prevalence of headaches, among others, in recent years [3,20,21,22,23,24,25]. For Germany, the prevalence of recurrent pain is assessed by the nationally representative German Health Interview and Examination Survey for Children and Adolescents (KiGGS) within the framework of health monitoring at the Robert Koch Institute (RKI) [26].

In the KiGGS baseline study (2003–2006), more than half of children and adolescents aged 11 to 17 years reported recurrent pain [6]. Headaches, abdominal pain, and back pain were the most frequently mentioned pain locations [6]. The prevalence was higher in girls compared with boys and in the older group (14–17 years) compared with the younger group (11–13 years) [6]. While only minor changes in prevalence were observed in KiGGS Wave 1 (2009–2012) [9], the following KiGGS Wave 2 (2014–2017) revealed significant increases in the prevalence of recurrent headaches, among other pains [27]. The relation with sociodemographic variables was investigated, but neither stratification by socioeconomic status (SES) nor migrant background revealed consistent differences [9].

Due to the fact that sustainable measures can only be planned and well-founded decisions made on the basis of current data and regular surveillance, one objective of the present study was to update the prevalence of recurrent pain in children and adolescents in Germany based on a current sample of the Motorik-Modul Study (MoMo) Wave 3 (2018–2020) and to compare it with the prevalence reported in previous surveys (i.e., KiGGS Wave 2). Furthermore, the present study assessed the association between PA and recurrent headaches, which, as shown by the previous studies, are the most common type of pain in children and adolescents in Germany. Therefore, PA is captured via accelerometry and evaluated based on the recommendation of the World Health Organization (WHO). Regarding the potential mechanisms linking enhanced cardiovascular fitness and migraines, we would expect an active lifestyle to lower the odds of recurrent headaches. Additionally, the study examined whether self-reported pain in the past three months reduces device-based measured MVPA levels. 

## 2. Materials and Methods

### 2.1. Procedure and Participants

The KiGGS core survey is supplemented by different in-depth modules [28]. One of these in-depth modules is the Motorik-Modul Study (MoMo) [29]. The main objectives of MoMo are to analyze the developmental and temporal trends in motor performance (MP), physical fitness (PF), and physical activity (PA), as well as their underlying influencing factors [29,30]. In addition, MoMo assesses the impact of MP/PF and PA on the health development of children and adolescents [29,30]. The selection for the representative cohort of MoMo Wave 3 was conducted according to the established procedure of the former waves. At the first level, the municipalities of the former waves were retained. In the second step, a stratified sample was randomly selected from the addresses submitted. More information about the sampling procedure can be found elsewhere [28]. The selected persons were invited to participate in the study by letter. Participation in the study was voluntary, and written consent was obtained prior to data collection. Under the age of 15, the participants had to be accompanied by a legal guardian. Each participant received an incentive after participating. 

The MoMo Wave 3 data collection started in August 2018 and was scheduled to end in June 2020. Due to the COVID-19 lockdown in Germany, MoMo Wave 3 had to be interrupted in March 2020. Therefore, only data collected between August 2018 and March 2020 were used. All tests were carried out by trained personnel. 

In total 2,843 participants (48.3% female) were enrolled in MoMo Wave 3. The preliminary response was 25.2% [31]. This results in a sample of 1516 participants, consisting of 770 girls (age = 14.5 ± 2.0 y., MVPA = 34.8 ± 18.3 min/day) and 746 boys (age = 14.3 ± 2.0 y., MVPA = 39.7 ± 20.1 min/day).

The STROBE statement guided the reporting of this study [32].

### 2.2. Measures and Variables

Except for the device-based measured PA, the data in the current study were self-reported by the participants and collected through a set of questionnaires completed on a PC or online. For information on the conception and the background of the *MoMo-PA-Questionnaire* (MoMo-AFB) see Schmidt et al. [33]. The reliability and validity of the MoMo-AFB are comparable to other international questionnaires [34]. 

#### 2.2.1. Demographic Information

Participants were asked for their sex and date of birth. The completed years of life on the day of the examination were used as the participant’s age in years.

#### 2.2.2. Pain

Self-reported pain in the last three months prior to the survey was captured by questionnaire. To ensure comparability with data from previous surveys, the questions about pain were adopted from the KiGGS Wave 2 questionnaire [27,35]. Participants received a list of various pain locations and indicated whether pain had occurred once, recurred, or not occurred in the past 3 months. The list of pain locations included: head, abdomen, back, ears, eyes, lower abdomen, arms/hands, legs/feet, throat, teeth, and thorax. Female participants were also asked about menstrual pain. For the analysis, the responses were dichotomized: pain occurred recurrently vs. no pain occurred or pain occurred only once (see also [27]). 

Prevalence was reported for all pain types asked in the questionnaire, while further analysis focused on recurrent headaches, recurrent abdominal pain, and recurrent back pain. In addition to the individual pain variables for the three localizations, a combined dichotomous variable was formed. This variable was set to true as soon as recurrent pain was reported for at least one of the three localizations.

#### 2.2.3. Data Selection

Due to the fact that for participants aged 4 to 10 years the questionnaire and thus the questions about pain were answered by parents, we included only data from participants aged 11 to 17 years in the analysis to avoid bias from possible parental assessment.

#### 2.2.4. Physical Activity

After completion of the PF/MP field tests, participants were asked to wear an accelerometer (GT3X+/wGT3X-BT, ActiGraph) on the right hip during their waking hours for the following week. Before further analyses, the non-wear time was removed from the data using an algorithm implemented by Choi et al. [36]. All days with wear time of 8 h or more after the removal of the non-wear time were considered valid days. Participants needed four or more valid weekdays and at least one valid day of the weekend to be included in the further analyses. The average time (min/day) spent in MVPA was derived from the accelerometer for all valid data sets. Participants who averaged at least 60 min of MVPA per day were considered active according to the WHO recommendation [37].

### 2.3. Data Analysis

The prevalences of different types of recurrent pain were calculated as the proportion of participants with pain in relation to the total number of participants in the group of interest. The prevalence of each type of pain is reported with 95% confidence intervals (95% CI) for girls and boys separately.

Logistic regression was used to test whether pain could be predicted based on sex, age, and activity status. 

Welch tests were used to compare the group means of the PA of participants with pain and those without pain. The Welch test has statistical power comparable to the t test when variances are equal, but unlike the latter, it is robust to unequal variances and skewed distributions [38]. This eliminates the need for a preliminary check of the test assumptions [38]. Multiple Welch tests were used to determine the differences between groups for different pain locations. Due to known differences in MVPA levels in girls and boys, the differences between participants reporting recurrent pain and those who did not were also analyzed separately for both sexes in all analyzes. 

The level of significance was set to 0.05 for all statistical analyses. The analyses were performed using R statistical software (v4.1.0, [39]).

## 3. Results

### 3.1. Prevalence of Pain

Table 1 and Table 2 show the prevalence rates of the pain locations surveyed in decreasing order for girls and boys, respectively. 

The prevalence rates for headache, abdominal pain, back pain, and lower abdominal pain were higher in girls than in boys. Heads, abdomens, and backs were among the most common locations of recurrent pain for both girls and boys.

### 3.2. Meeting the WHO PA Recommendation and Pain

The logistic regression model for headaches (*X2*(2) = 13.03, *p* = 0.001, R²(Nagelkerke) = 0.029) was significant, and it did not improve by including age. The ORs of the final model are presented in Table 3.

### 3.3. Pain and Physical Activity

In boys, Welch tests did not show significant differences in device-based MVPA between those who reported recurrent headaches and those who did not (M_diff_ = 2.4 min, 95% CI [−2.7, 7.4], t(157.75) = 0.93, *p* = 0.355). There were also no differences between the boys that reported recurrent abdominal pain (M_diff_ =4.5 min, 95% CI [−0.7, 9.7], t(95.13) = 1.72, *p* = 0.088) or back pain (M_diff_ = 3.0 min, 95% CI [−3.5, 9.5], t(76.1) = 0.92, *p* = 0.359) and those who did not.

Girls who reported recurrent headaches in the last three months averaged 4.7 min (95% CI [0.74, 8.66], t(297.29) = 2.33, *p* = 0.020) less MVPA than girls who reported having no headaches. Among girls who reported recurrent back pain, MVPA was 5.8 min (95% CI [1.58, 10.04]) lower on average (t(156.63) = 2.71, *p* = 0.007). For abdominal pain, the difference in device-based MVPA was not significant (M_diff_ =−1.3 min, 95% CI [−5.5, 3.0], t(171) = −0.58, *p* = 0.561).

## 4. Discussion

The present study aimed to describe the prevalence of recurrent pain in a German sample of children aged 11–17 years and to assess whether PA is associated with the occurrence of recurrent pain in children and adolescents. 

We found differences between girls and boys in the prevalence of recurrent pain in several locations. Recurrent pain was observed more frequently in girls than in boys for headaches, abdominal pain, back pain, and lower abdominal pain. The most pronounced difference observed was for headaches. The analysis showed increased odds of recurrent headaches if the analyzed participant was a girl or if the participant failed to achieve the recommended average of 60 min of MVPA per day. We only found reduced PA levels in girls reporting recurrent pain, but not in boys. 

For the most part, the prevalence observed in the present study did not differ from that reported in KiGGS Wave 2 [27]. Only the prevalence of recurrent abdominal pain in girls’ pain was higher (KiGGS Wave 2: 34.5%, 95% CI [31.9, 37.2]) than in the present study (MoMo Wave 3: 28.2%, 95% CI [25.1, 31.5]). Regarding this, we have to keep in mind that the survey in the KiGGS study was supplemented by medical examinations that do not require physical exertion on the part of the participant, while a considerable part of MoMo consists of motor test tasks. Therefore, it is possible for those, especially girls with acute complaints due to menstrual or abdominal pain, to refrain from participating in the latter. All in all, the prevalence of recurrent headaches and back pain in children and adolescents in Germany seems to remain constant on a high level over the last 5 to 10 years. Especially with regard to recurrent headaches, it seems important to take action against the high prevalence, as previous studies have shown that headaches in adolescents leads to significant impairments in HRQoL [24,40]. The fact that recurrent headaches were the most frequently cited pain in both sexes in the present data underscores the urgency of action. At the same time, the current figures indicate that either no steps have been initiated so far or that previous steps have not led to an improvement compared with the figures from KiGGS Wave 2 (2014–2017).

The Norwegian HUNT study found that recurrent headaches were associated with an unfavorable lifestyle among adolescents. The study addressed being overweight and low PA, among other factors [19]. Their analysis showed an elevated OR of recurrent headaches for overweight (OR = 1.4, 95% CI [1.2, 1.6], *p* < 0.001) and low PA (OR = 1.2, 95% CI [1.1, 1.4], *p*= 0.002) adolescents [19]. The association with being overweight was not tested in the current analysis, but higher odds were also found for the low-active group (OR (low PA) = 2.06, 95% CI [1.1, 4.17], *p* = 0.001). Different definitions of the threshold for low activity or the underlying metric probably explain the different magnitudes, but the direction of the effect shows a consistent picture that PA could lower the odds of recurrent headaches. The association of headaches with an unhealthy lifestyle has also been observed in other studies [41,42,43]. The lack of PA was often used as part of an expression of an unhealthy lifestyle. Therefore, the lack of PA showed an increased risk of migraines (OR = 4.2, 95% CI [2.2, 7.9]) and tension-type headaches (OR = 1.7, 95% CI [1.1, 2.7]) [42]. In addition, social stress factors such as family conflicts, school, or bullying experiences have also been identified as possible causes of headaches [44,45]. The current results underline the importance of promoting a healthy lifestyle in children and adolescents, including adequate levels of physical activity. In addition, further steps may be also beneficial, e. g., to reduce stress in the family or at school.

The difference in the prevalences of recurrent pain between girls and boys that was found in our analyses is in line with the results of KiGGS [9,27] and several international studies [23,24,46]. Various reasons are discussed as explanations. On the one hand, it is assumed that girls perceive their bodies differently than boys, and that they are more sensitive and more willing to communicate their feelings [47]. On the other hand, different pain processing in the brain and hormonal differences could play a role [48,49].

In our results, we found lower MVPA in girls with recurrent headaches or back pain compared with those who reported no pain. This suggests that the presence of pain may have a negative impact on PA, which in turn would support the findings of another population-based survey that also reported reduced MVPA in the presence of pain [50]. 

In summary, the results confirm the high prevalence rates reported in previous surveys of German youth. Logistic regression shows that only a small part of the variance in our sample can be explained by the factors examined. At the same time, it should be kept in mind that both physical activity and pain are very complex constructs. However, the results show a consistent picture and indicate that being female and having a low activity status are associated with a higher prevalence of recurrent pain, especially headaches and abdominal pain. 

One strength of the MoMo study data set is its representative sample [28,29]. Although data collection in the current wave ended prematurely due to the pandemic, data was collected in about two-thirds of the test sites according to the preestablished protocol. On the other hand, the MoMo study uses proven methods on the different waves, which allows for comparability between the different waves of the study. 

Nevertheless, there are some limitations to consider. The representativeness of the sample is compromised by the discontinuation of the study due to the pandemic. Due to the fact that the results of the study are on the same level as those of KiGGS Wave 2, it can be assumed that the impact due to early termination is manageable. Pain data were collected by questionnaire, and therefore may be biased by recall error or incorrect information due to social desirability [51,52,53]. In particular, with regard to the questions on pain, it can be seen that the recall period of three months is relatively long. To prevent misperceptions of health statuses by parents from becoming a problem, only data from children 11 years and older were analyzed. From this age, the participants fill out the questionnaire themselves. In addition, it is possible that children and adolescents currently suffering from pain may not participate in the study at all or refuse to wear the accelerometer, resulting in exclusion from the analysis due to missing data.

Last but not least, based on the cross-sectional data, we can only make statements about the correlations, but cannot draw conclusions about causal effects.

## 5. Conclusions

The prevalence of pain, especially of recurrent headaches, abdominal pain, and back pain, remains at a high level in girls as well as in boys in Germany, and therefore underscores the need for interventions to improve the health situation of children and adolescents. First, more research is needed to find the reasons for such a high pain prevalence while also considering sex differences in frequencies and types of pain. Lifestyle recommendations play an important role in the treatment and prevention of pain in childhood, and especially in adolescence [54]. Therefore, it is important to further investigate the effects of lifestyle factors, such as PA, to gain a better understanding and therefore make more targeted recommendations. This also needs longitudinal data to investigate causal effects between PA and pain. Furthermore, it should be considered that PA, in addition to the presumed preventive effect against pain, can also be a trigger of the same. Finally, the presence of pain can influence PA levels, and thus should therefore be considered when assessing PA to avoid bias. 

## Figures and Tables

**Table 1:** Prevalence and 95% CI of recurrent pain in girls.

Pain Location	N	Prevalence (in %)	Lower 95% CI (in %)	Upper 95% CI (in %)
**head**	756	42.7 *	39.2	46.3
**menstrual pain ^1^**	510	41.4	37.2	45.7
**abdomen**	752	28.2 *	25.1	31.5
**back**	755	26.9 *	23.8	30.2
**legs/feet**	613	21.9	18.8	25.3
**lower abdomen**	614	20.0 *	17.1	23.4
**throat**	614	12.7	10.3	15.6
**arms/hands**	612	8.5	6.5	11.0
**thorax**	751	5.5	4.0	7.3
**teeth**	611	5.2	3.7	7.3
**eyes**	614	4.1	2.8	5.9
**ears**	613	2.3	1.4	3.8

* different from prevalence in boys, *p* < 0.05, ^1^ menstrual pain was only analyzed for girls.

**Table 2:** Prevalence and 95% CI of recurrent pain for boys.

Pain Location	N	Prevalence (in %)	Lower 95% CI (in %)	Upper 95% CI (in %)
**head**	729	28.7 *	25.5	32.1
**legs/feet**	612	22.7	19.6	26.2
**abdomen**	728	20.1 *	17.3	23.1
**back**	728	19.5 *	16.8	22.5
**throat**	613	11.6	9.3	14.4
**arms/hands**	612	7.5	5.7	9.9
**thorax**	724	5.1	3.7	7.0
**teeth**	612	4.7	3.3	6.7
**ears**	614	2.1	1.2	3.6
**eyes**	614	3.4	2.2	5.2
**lower abdomen**	708	1.6 *	0.9	3.0

* different from prevalence in girls, *p* < 0.05.

**Table 3:** Logistic regression model for recurrent headaches.

	OR (95% CI)	*p*
**(Intercept)**	0.21 (0.11–0.39)	<0.001
**Sex**		
**Male**	Ref.	
**Female**	1.54 (1.09–2.18)	0.014
**Meeting the recommendation**		
**Yes**	Ref.	
**No**	2.06 (1.1–4.17)	0.033

## Data Availability

Original data can be derived from the corresponding author on request.
